# Translaminar Screw Fixation for Giant C1 Lateral Mass Metastasis From Hepatocellular Carcinoma

**DOI:** 10.7759/cureus.81062

**Published:** 2025-03-24

**Authors:** Ryo Sakisuka, Hideki Hayashi, Yoshito Sugita, Hirokuni Hashikata, Hiroki Toda

**Affiliations:** 1 Department of Neurosurgery, Shinko Hospital, Kobe, JPN; 2 Department of Neurosurgery, Medical Research Institute Kitano Hospital, Public Interest Incorporated Foundation (PIIF) Tazuke-Kofukai, Osaka, JPN

**Keywords:** craniovertebral junction instability, hepatocellular carcinoma (hcc), metastatic spinal tumor, posterior fixation surgery, translaminar screw

## Abstract

Metastatic spinal tumors at the craniovertebral junction (CVJ) are exceedingly rare, with limited reports of C1 lateral mass metastases from hepatocellular carcinoma (HCC). This report presents a case in which posterior fixation alone successfully achieved pain relief and improved stability in a patient with HCC metastasis involving extensive osteolysis of a C1 lateral mass and encasement of the vertebral artery.

A 71-year-old male presented with a worsening right cervical pain. The patient had been prescribed nonsteroidal anti-inflammatory drugs and opioid analgesics, yet the pain remained unresolved. Computed tomography (CT) revealed a 5.5 cm osteolytic tumor at the C1 lateral mass with circumferential involvement of the vertebral artery. The patient was diagnosed with spinal metastasis from HCC, characterized by spinal instability (Spinal Instability Neoplastic Score (SINS), 11) and intractable pain (visual analog scale (VAS), 7.7). The patient was bedridden because of an inability to support head loads (performance status (PS), 4), and it was considered difficult to initiate ongoing chemotherapy. The patient was referred for surgical intervention to relieve the pain and improve stability. To address the pain and instability, occipitocervical (C2-C5) posterior fixation was performed without tumor resection. Due to extensive tumor invasion, pedicle screw placement at C2 was not feasible, and C2 translaminar screw fixation was selected instead. Postoperatively, the patient experienced significant pain relief and regained ambulatory ability. At one month postoperatively, fixation remained stable, with VAS improving to 0.8 and PS to 1. The patient was able to maintain pain relief and exhibited improved stability, allowing him to walk, and durvalumab/tremelimumab chemotherapy was initiated two months after surgery.

The primary goals of managing metastatic tumors at the CVJ are pain relief, neurological function preservation, and spinal stability. In this case, posterior fixation alone achieved substantial pain alleviation and stability improvement without tumor resection. Notably, the use of C2 translaminar screws minimized the risks of spinal cord and vertebral artery injuries while ensuring effective stabilization. This is the first report to demonstrate the efficacy of posterior fixation alone for HCC metastases with complete destruction of the articular facet at C1.

Posterior fixation alone can provide effective pain relief and restore stability in metastatic tumors with severe osteolysis of the C1 lateral mass. Careful selection of fixation techniques can minimize intraoperative complications while improving the patient’s quality of life. This report contributes to surgical strategies for similar cases.

## Introduction

Metastatic spinal tumors at the craniovertebral junction (CVJ) are rare, accounting for approximately 0.5% of all spinal metastases [1]. However, with the advancements in cancer treatment, recent reports on treatment strategies have emerged. Most metastatic spinal tumors of the CVJ present with neck pain and neurological symptoms [2]. According to the 2024 recommendations of the World Federation of Neurosurgical Societies (WFNS) Spine Committee, surgical intervention, particularly occipitocervical posterior fixation, is recommended [3]. Posterior fixation alone has been reported to restore stability and provide pain relief [4]. A retrospective cohort study reported that occipitocervical posterior fixation with C2 pedicle screws effectively relieved the neck pain [5].

Reports of hepatocellular carcinoma (HCC) metastasizing to the CVJ remain scarce [2,6], and no cases have been documented in which posterior fixation was performed for a giant tumor causing complete destruction of the atlanto-occipital joint, including damage to the anterior arch and the transverse ligament.

Herein, we present a case of a giant metastatic tumor from HCC in the right C1 lateral mass that caused complete osteolysis of the articular cavity and circumferential involvement of the vertebral artery. Occipitocervical posterior fixation with C2 translaminar screws was performed to achieve effective pain relief and restoration of stability.

## Technical report

**Figure 1:**
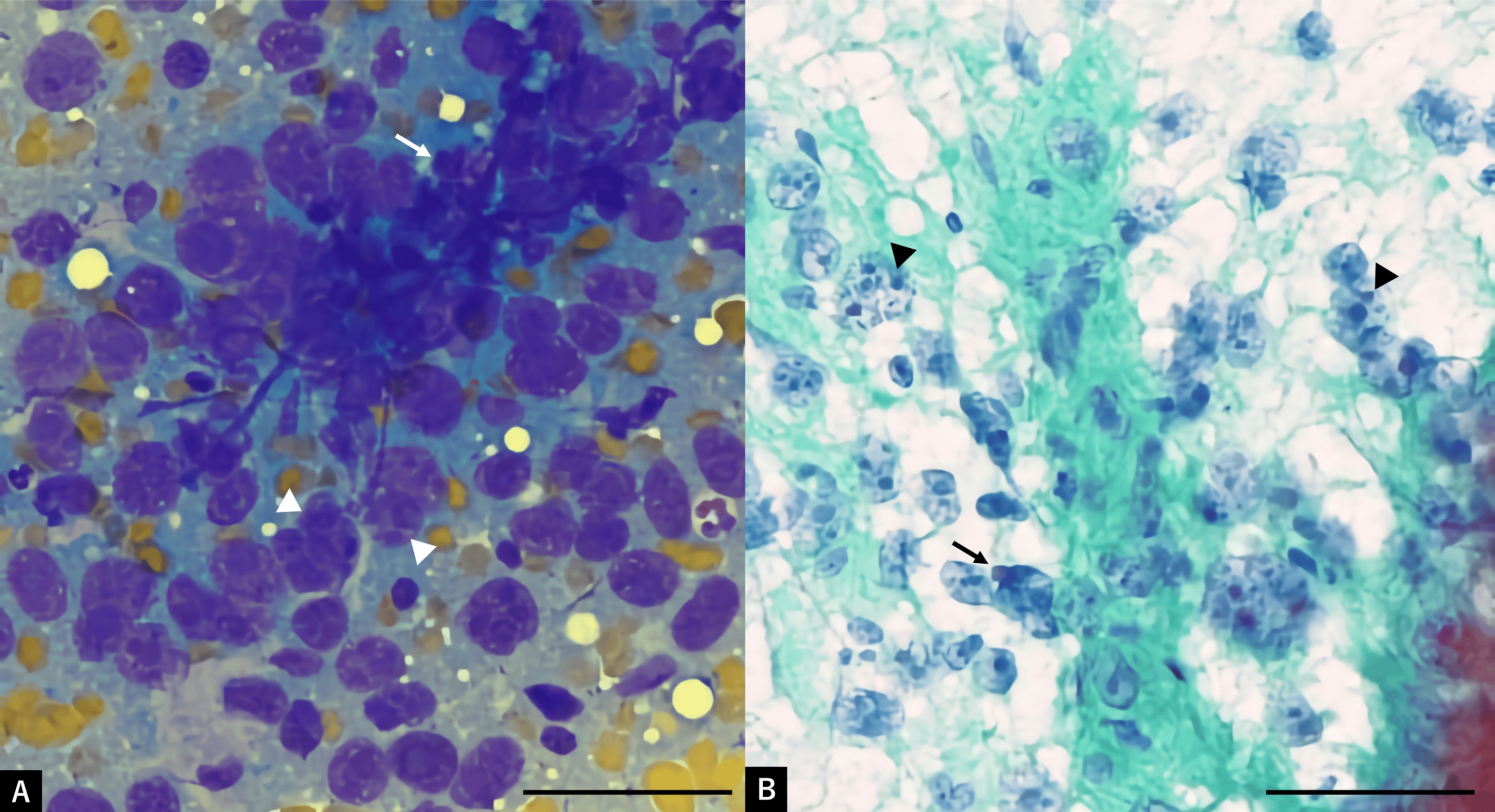
Pathological examination of fine needle aspiration cytology (A) Giemsa staining (×400). (B) Papanicolaou staining (×400). Clusters and isolated highly atypical cells are extensively observed. Marked pleomorphism, nuclear irregularity, and prominent nucleoli (arrowhead) were evident, with most cells appearing as isolated naked nuclei (arrow). Scale bar = 100 μm.

A 71-year-old male presented with right-sided neck pain, persisting for two months, which progressively worsened with difficulty in maintaining a sitting position. The patient had comorbidities, such as hypertension, diabetes, and early-stage gastric cancer. The early-stage gastric cancer was completely cured by endoscopic submucosal dissection, and he had no other surgical history. No family history was noted. Plain computed tomography (CT) showed a tumor on the right side of the CVJ. He was initially suspected of having hypoglossal schwannoma by a local physician and was referred to our hospital. Three weeks later, an endoscopic ultrasound-guided biopsy of a tumor on the right side of the CVJ performed by the otolaryngology department revealed a malignancy (Figures 1A-1B).

**Figure 2:**
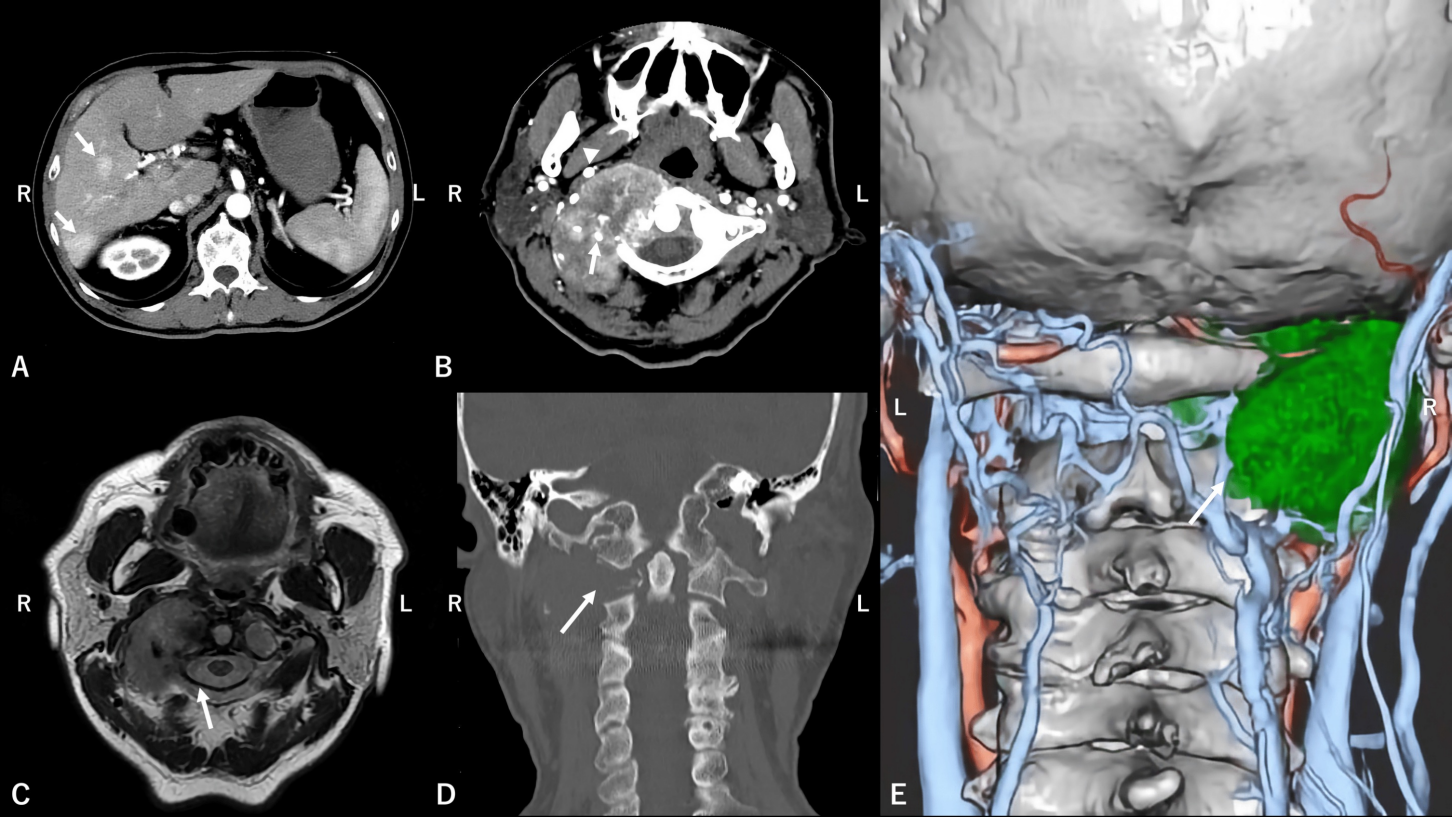
Preoperative imaging findings (A) Dynamic contrast-enhanced abdominal CT: Multiple liver masses (arrow) exhibiting arterial-phase enhancement and lack of enhancement in the portal venous phase, leading to a diagnosis of primary hepatocellular carcinoma (HCC). (B) Contrast-enhanced cervical CT: A 5.5 cm lytic tumor is identified in the right C1 lateral mass, extending to the anterior and posterior arches and involving the paravertebral muscles. The right vertebral artery (arrow) is completely involved, and the internal carotid artery (arrowhead) is anteriorly compressed by the tumor. (C) Plain cervical MRI T2 weighted image: An extradurally extending tumor of the right C1 lateral mass is observed, but there is no compression or deformation of the spinal cord (arrow). (D) Plain cervical CT showing complete destruction of the right C1 lateral mass (arrow), suggesting severe instability. (E) Reconstructed enhanced CT: The tumor extends to the posterior aspect of the C2 lamina (arrow), rendering pedicle screw placement unfeasible.

Serological tests showed positive HBs and HBc antibodies, markedly elevated AFP (36,093.6 ng/mL), and Protein Induced by Vitamin K Absence (PIVKA)-II (2,168 mAU/mL). Contrast-enhanced abdominal CT revealed multiple hypervascular liver tumors (Figure 2A), leading to a diagnosis of primary HCC. The patient had preserved liver function (Child-Pugh score of 5, class A). CT demonstrated osteolytic lesions in the right ilium and right C1 lateral mass (Figure 2B), and magnetic resonance imaging (MRI) confirmed similar signal changes in the liver, right C1 lateral mass, and right ilium. A report on fine-needle aspiration (FNA) for metastatic HCC described the following cytological characteristics: single tumor cells (88.9%), cytoplasmic vacuolation (70.4%), trabecular architecture (70.4%), naked nuclei (66.7%), and prominent nucleoli (66.7%) [7]. The findings in this case were consistent with those of spinal metastases from HCC. The patient was bedridden because of an inability to support head loads (performance status (PS), 4), making it difficult to initiate ongoing chemotherapy in an outpatient gastroenterology department. The patient was referred to our department for surgical intervention aimed at pain relief and exhibited improved stability.

On admission, he exhibited right vagus and hypoglossal nerve palsy, along with severe neck pain (visual analog scale (VAS) 7.7). He had been prescribed non-steroidal anti-inflammatory drugs (NSAIDs) and opioid analgesics, yet the pain remained unresolved. Cervical CT showed a 5.5 cm osteolytic tumor in the right C1 lateral mass with circumferential involvement of the right vertebral artery, while the right internal carotid artery and internal jugular vein were anteriorly displaced (Figure 2B). The Epidural Spinal Cord Compression (ESCC) scale score was 1, with no apparent spinal cord compression (Figure 2C). The tumor completely osteolyzed the C1 lateral mass and extended into the dorsal C2 lamina of the vertebral arch (Figures 2D-2E). Contrast-enhanced CT/MRI suggested a hypervascular tumor.

The Spinal Instability Neoplastic Score (SINS) was 11, indicating significant instability. The modified Tokuhashi score was 8, suggesting a prognosis of less than six months, whereas the Tomita score was six, predicting a six to 12 months of prognosis. Furthermore, a gastroenterologist estimated that if chemotherapy was to be administered, the patient's survival prognosis would exceed six months. It is crucial to address instability through surgical intervention, enhance PS, and initiate chemotherapy. Based on these assessments, surgical intervention was prioritized over palliative radiation therapy.

**Figure 3:**
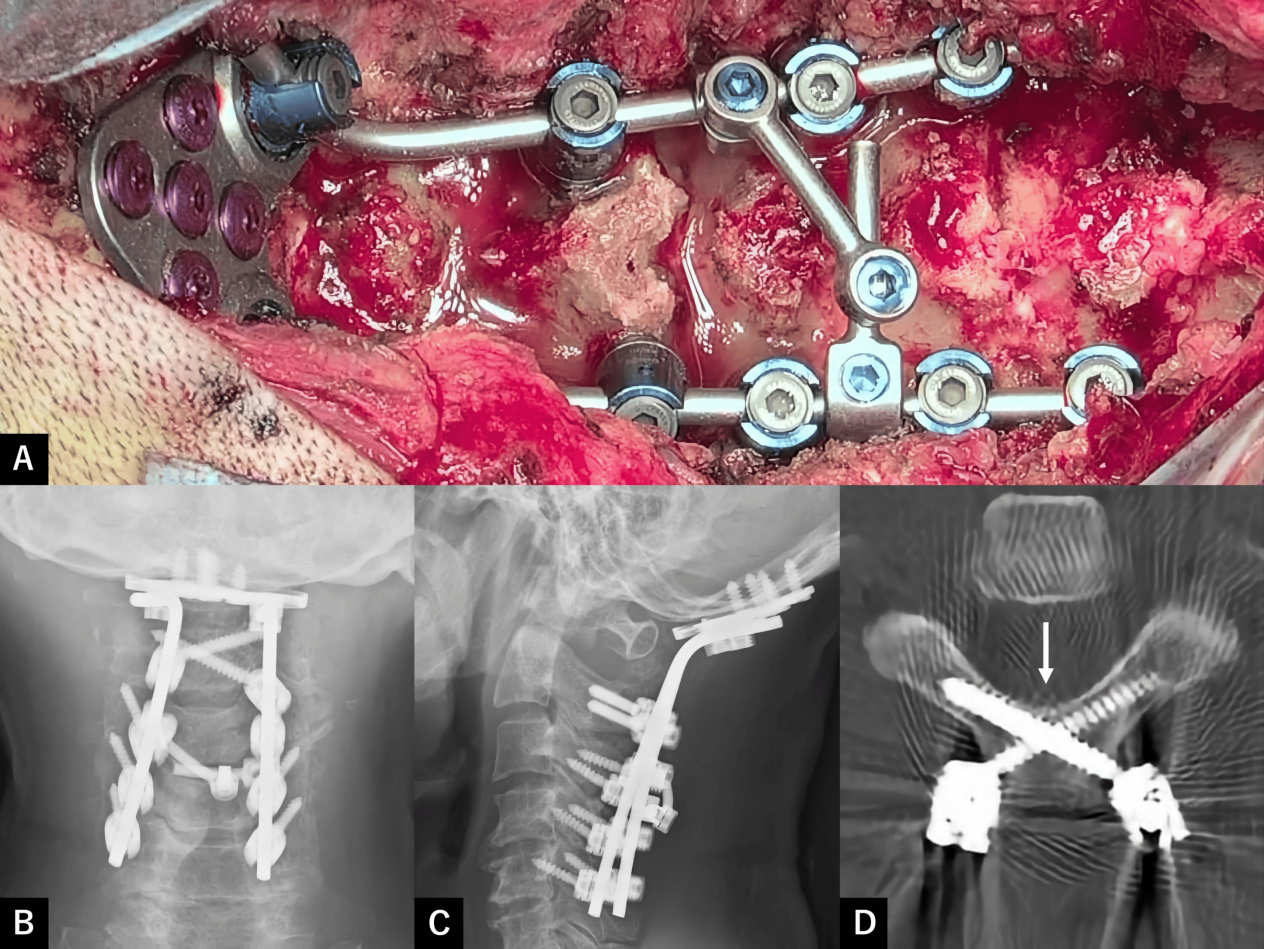
Intraoperative photograph and imaging findings at one month after surgery (A) Intraoperative photograph. (B) Anteroposterior cervical X-ray. (C) Lateral cervical X-ray. (D) Axial section of cervical CT. We performed occipito-C2-C5 posterior fixation, which included the insertion of C2 translaminar screws and C3-C5 lateral mass screws. One month after surgery, imaging studies confirmed the stability of the fixed screws and rods. Cervical CT showing bilateral C2 translaminar screws (arrow).

Due to the high risk of hemorrhage, tumor resection was not performed. Instead, posterior fixation was performed to relieve pain and improve spinal instability. Given the complete osteolysis of the unilateral articular cavity and resultant severe instability, occipitocervical (C2-C5) posterior fixation was planned. Surgery was performed under C-arm fluoroscopic guidance without intraoperative navigation. A midline incision was made from the inion to C7 in the prone position. The occipital bone was secured using an occipital adjustable plate fixed with 4.5 mm diameter titanium screws. Due to tumor extension to the posterior C2 lamina, pedicle screw insertion was not feasible; therefore, translaminar 4.5 mm diameter titanium screws were inserted instead. Lateral mass 3.5 mm diameter titanium screws were placed in C3-C5 and secured with pre-cut/bent 3.5 mm diameter titanium rods and a rod crosslink. The spinous process was resected, crushed, and grafted onto the facet joints and occipital bone surface before wound closure (Figure 3A). The operation time was five hours and 33 minutes, with a blood loss of 148 mL.

Postoperatively, the patient experienced significant pain relief and was able to walk. External beam radiation therapy (3 Gy/10 fractions) was initiated on postoperative day 16. At one month postoperatively, VAS improved to 0.8, PS improved to 1, and cervical CT confirmed stable screw placement and proper spinal alignment (Figures 3B-3D). The patient was discharged on postoperative day 31. At present, two months postoperatively, the patient has maintained pain relief and stability, allowing him to walk. Hoarseness due to vagus nerve paralysis has slightly worsened, and hypoglossal nerve paralysis remains unchanged; however, the patient can speak and eat without difficulty. Durvalumab/tremelimumab chemotherapy was initiated because of an improvement in PS.

## Discussion

Management of metastatic tumors at the craniovertebral junction

Primary symptoms of CVJ metastatic tumors include pain during movement and occipital neuralgia. Rotational pain and occipital neuralgia occurred in 90% and 33% of the patients, respectively. Myelopathy due to spinal cord compression is relatively rare (0-22%) [8]. C2 was the most commonly affected vertebra (83%), and among C1 metastases (31%), lateral mass involvement was predominant (88%) [9]. The treatment goals for metastatic CVJ tumors include pain relief, improvement in neurological function, and maintenance of spinal stability. Surgical indications included PS assessment, prognosis estimation, systemic metastasis evaluation, and the presence of effective systemic therapy options. Patients with poor PS and a prognosis of fewer than two months, severe visceral metastases with poor surgical tolerance, or uncontrolled systemic diseases are generally excluded from surgery. However, reports indicate that even in cases with poor Tokuhashi scores (zero to eight points), survival is significantly prolonged if effective systemic therapy is available [10]. Surgical intervention is recommended in cases of significant instability (SINS ≥7), progressive neurological deficits, intractable pain, or radiation therapy exceeding spinal cord tolerance [3]. Even with moderate instability (SINS 7-12), surgery is advocated in the presence of pain [9]. The preferred approach is occipito-C2 posterior fixation [3], which effectively restores stability and alleviates pain without tumor resection [8]. Similarly, long-term mechanical stability has been observed with pars, pedicle, and translaminar screws [4]. Screw fixation is reportedly superior to wire fixation in terms of pain relief and fixation strength [11]. In cervical tumor surgery, the presence of C1-2 tumors, combined anterior-posterior approaches, and prolonged operative time (>3 hours) are independent predictors of complications, such as neurological deficits, prolonged tracheal intubation, pneumonia, and vertebral artery injury [12]. Posterior transpedicular corpectomy and cervical vertebroplasty are novel surgical techniques currently reported only in case studies, and further long-term evaluation of their outcomes is necessary [3]. 

In the present case, the patient was eligible for postoperative systemic treatment, except for PS, and underwent surgery due to spinal instability (SINS 11) and intractable pain. The mass effect of the tumor caused right vagal and hypoglossal paralysis; however, no spinal symptoms were observed. Since HCC was clinically diagnosed, partial tumor removal was not performed for diagnostic purposes. In addition, partial removal of the tumor through a posterior approach was considered insufficient because most of the tumor needed to be removed to relieve pressure on the vagus and hypoglossal nerves, which were compressed ventrally by the tumor. The tumor was hypervascularized, completely involving the vertebral artery and abutting the internal carotid artery and internal jugular vein ventral to the tumor, making an anterolateral approach to remove the tumor highly likely to cause hemostasis difficulties and damage to these vessels. In addition, the primary goal is to relieve pain and improve spinal stability to improve PS and enable chemotherapy. Therefore, tumor resection via an anterolateral approach was not performed.

C2 translaminar screw fixation

C2 translaminar screws offer a low risk of spinal cord and vertebral artery injuries and can be placed without intraoperative navigation [13]. These screws pose minimal risk to neural and vascular structures while providing stability equivalent to C2 pedicle screws. A previous study reported successful fixation in 97.6% of cases without vertebral artery injury [14]. However, C2 translaminar screws may have inferior resistance to lateral bending forces compared to other screw types if the atlantoaxial ligaments are damaged [14]. The C2 lamina is generally thicker and broader in males than in females, and fixation is often challenging in children under six years of age [15]. In this case, 4.5 mm diameter translaminar screws were successfully inserted without injuring the hypervascular tumor. One month postoperatively, the screws remained intact and stable fixation was maintained.

Management of spinal metastases from hepatocellular carcinoma

HCC is the third leading cause of cancer-related death worldwide. The introduction of atezolizumab and bevacizumab has led to improved survival rates [16]. HCC metastases are typically osteolytic, with an estimated incidence of spinal metastases ranging from 1.5% to 7.3% [17]. The distribution of metastatic lesions includes 14.5% of the cervical spine, 44.7% of the thoracic spine, 22.4% of the lumbar spine, and 3.9% of the sacrum. Cervical metastasis is relatively rare [18]. The overall survival of patients with spinal metastases from HCC is 10.6 months. The median survival time was 12 months for the Child-Pugh class A patients and five months for the class B/C patients. Patients with a Tomita score of ≥8 have a median survival of eight months, whereas those with a score of ≤7 have a median survival of 23.8 months [18]. HCC is considered radioresistant, with an 87% pain relief rate but relatively low local control rates at six months (61.6%) and 12 months (40.8%) [19]. Surgical fixation is necessary in patients with spinal instability. Surgical treatment has been associated with a significant improvement in overall survival (10.46 ± 8.05 months vs. 5.19 ± 7.72 months) and significantly improved activities of daily living, whereas paralysis progression was significantly worse in the conservative treatment group [20]. The article stated that 13 patients in the surgical group underwent posterior decompression and fixation, while 12 patients in the surgical group underwent posterior fixation alone. However, a notable omission in the study was a comparison between the groups that underwent decompression and those that did not, and the superiority of tumor removal was not demonstrated. It is also mentioned that one patient died of liver failure within the first postoperative month after excessive intraoperative bleeding despite strict coagulation factor therapy. A case-by-case approach is warranted to determine the necessity for tumor removal.

According to the Japanese Guidelines for the Treatment of Liver Cancer [21], chemotherapy is recommended for patients with extrahepatic metastases, preserved hepatic reserve (Child-Pugh A), and good PS (PS = 0 or 1). Additionally, in Japan, patients are not typically hospitalized for continued intravenous chemotherapy because of insurance reimbursement policies. Instead, oral chemotherapy is primarily administered in outpatient settings. 

In this case, the patient was classified as Child-Pugh class A, with a Tomita score of six, indicating a prognosis of ≥6 months. However, the patient was bedridden due to severe neck pain and instability, and chemotherapy could not be initiated. Radiation therapy has been demonstrated to be effective in alleviating pain; however, it does not improve instability. Therefore, by improving his PS through surgical intervention, chemotherapy became feasible postoperatively. Since both the right occipito-atlas and atlanto-axial joints were destroyed and relatively long-term survival was expected, the range of fixation was extended to C5 for long-term stability. 

Reports and treatment of C1 metastasis from hepatocellular carcinoma

A PubMed search using the terms “metastatic hepatocellular carcinoma”, “upper cervical”, “occipito-cervical junction”, and “cranio-vertebral junction” revealed only two previous reports of C1 lateral mass metastases from HCC. No prior reports have described a giant tumor extending from the hypoglossal canal and jugular foramen to the posterior C2 lamina nor have any technical notes on fixation methods. The first reported case involved metastases to the occipital bone, C1 lateral mass, and C5/6, treated with occipital tumor resection and occipito-C2/3/4 posterior fixation (C2: pedicle screw; C3/4: lateral mass screw). The patient died of liver failure 13 months after the surgery [6]. The second case involved an isolated C1 lateral mass metastasis treated with biopsy and occipito-C2/3 posterior fixation (screw-type unspecified), resulting in pain relief. However, the patient died three months postoperatively because of disease progression [2]. In the present case, the tumor extended to the posterior C2 lamina and prevented pedicle screw insertion. Therefore, translaminar screws were used. Given tumor size and instability, it is essential to select an appropriate fixation method and range.

Limitations

This report describes a single case; thus, the generalizability and universal applicability of this treatment strategy remain unclear. The follow-up period was short, necessitating further observation to assess the long-term stability and symptom progression due to tumor growth. However, the efficacy of tumor resection has not been evaluated. The relationship between fixation range and postoperative quality of life has only been analyzed in a retrospective case series. A shorter fixation range might have provided comparable stability, warranting the further accumulation of similar cases.

## Conclusions

Surgical treatment of metastatic spinal tumors at the CVJ aims to achieve pain management, spinal stabilization, and tumor control. In metastatic C1 lateral mass tumors, posterior fixation effectively improves instability and relieves pain. Even in cases of extensive articular facet destruction, posterior fixation alone can provide adequate pain relief, emphasizing the importance of selecting a fixation method that minimizes intraoperative complications, such as the translaminar screw used in this case. Early surgical intervention should be considered when the prognosis and overall treatment plan indicate suitability.
